# Characterization of PHB1 and Its Role in Mitochondrial Maturation and Yolk Platelet Degradation during Development of *Artemia* Embryos

**DOI:** 10.1371/journal.pone.0109152

**Published:** 2014-10-13

**Authors:** Xiang Ye, Yang Zhao, Ling-Ling Zhao, Yu-Xia Sun, Jin-Shu Yang, Wei-Jun Yang

**Affiliations:** 1 Key Laboratory of Conservation Biology for Endangered Wildlife of the Ministry of Education and College of Life Sciences, Zhejiang University, Hangzhou, Zhejiang, People's Republic of China; 2 Department of Molecular and Cell Biology, University of California, Berkeley, California, United States of America; Baylor College of Medicine, United States of America

## Abstract

**Background:**

To cope with harsh environments, crustaceans such as Artemia produce diapause gastrula embryos (cysts) with suppressed metabolism. Metabolism and development resume during post-diapause development, but the mechanism behind these cellular events remains largely unknown.

**Principal Finding:**

Our study investigated the role of prohibitin 1 (PHB1) in metabolic reinitiation during post-diapause development. We found that PHB1 was developmentally regulated via changes in phosphorylation status and localization. Results from RNA interference experiments demonstrated PHB1 to be critical for mitochondrial maturation and yolk degradation during development. In addition, PHB1 was present in yolk platelets, and it underwent ubiquitin-mediated degradation during the proteolysis of yolk protein.

**Conclusions/Significance:**

PHB1 has an indispensable role in coordinating mitochondrial maturation and yolk platelet degradation during development in *Artemia*. This novel function of PHB1 provides new clues to comprehend the roles of PHB1 in metabolism and development.

## Introduction

Prohibitin 1 (PHB1), a prohibitin family protein, forms a large complex with PHB2 at the inner mitochondrial membrane. PHB1 has emerged as a key regulator of several cellular events, including mitochondrial morphogenesis, cell adhesion, cell cycle regulation, and fat metabolism [1]. For example, PHB1 regulates transcription in the nucleus by interacting with E2F, retinoblastoma protein (Rb), and chromatin-remodeling complexes (Brg-1/Brm). Repression of E2F-mediated [3] transcription by PHB1 requires histone deacetylation and co-repressors (e.g., N-CoR) [2] [4] [5] [6]. PHB1 also inhibits DNA replication by interacting with members of Minichromosome maintenance complex of proteins (MCM2-7) proteins in the nucleus [7]. On the other hand, phosphorylation of PHB1 results in its association with the plasma membrane, activation of the Ras–Raf signaling pathway, which regulates epithelial cell adhesion and migration [8], and metastasis [9]. In light of these findings, PHB1 is a promising target for clinical applications because natural anticancer compounds such as rocaglamide can inhibit the Ras−Raf signaling pathway by binding to PHB1 [10].

Among the many emerging roles of PHB1, its role in the maintenance of mitochondrial integrity is perhaps the most important. At the mitochondrial inner membrane, PHB1 and PHB2 assemble into a large ring-like complex that stabilizes newly synthesized mitochondrial respiratory enzymes [11]. Prohibitins also function in cell proliferation and apoptosis by regulating the processing of the dynamin-like GTPase OPA1, thus control morphogenesis of mitochondrial cristae [12]. PHB1-mediated regulation of mitochondrial metabolism also links with cell senescence [13] [14]. For example, Marta et al. have reported that prohibitins promote longevity in *Caenorhabditis elegans* by affecting mitochondrial function and fat metabolism, which extends life span in certain genetic background [15]. The loss of prohibitin also impairs mitochondrial architecture and results in neurodegeneration in the mouse, which is indicative of aging [16].

PHB1 has many tissue-specific functions. It is strongly expressed by cells such as adipocytes, muscle cells, and β-cells that rely on efficient mitochondrial function and high energy levels. The loss of PHB markedly reduces fat content in adult nematodes and induces mitochondrial damages in β-cells of mammals, which results in the development of diabetes [15] [17]. In addition, PHB1 is critical for adipocyte differentiation, attenuating insulin-stimulated fatty acid and glucose oxidation in adipose tissue [18] [19] [20]. Thus, PHB1 plays an important role in age-related diseases by affecting metabolic processes; however, the molecular mechanism behind aberrant PHB1 function is not known.

PHB1 contains several highly conserved domains, namely, an N-terminal hydrophobic domain, a PHB domain, a coiled-coil domain, and a nuclear export sequence [2] [9] [21]. The N-terminal hydrophobic domain is critical for its attachment to the mitochondrial inner membrane, whereas the coiled-coil domain is important for protein–protein interactions [22]. The PHB domain is an integral membrane domain that is conserved from protozoa to mammals, and Tyr 114 and Ser 121 within this domain are phosphorylated and the O-GlcNAc modification of Ser 121 can be reduced by mutation of Tyr 114 to phenylalanine. Thus both residues have important roles in tyrosine kinase signaling pathways, including insulin, growth factors, and immune receptors signaling [23] [24].

*Artemia* (brine shrimp) are extremophiles that possess a unique mechanism of reproduction, that is, they are capable of laying encysted gastrula embryos (cysts) to cope with harsh environments such as anoxia, high salinity, high pH, and ion and temperature fluctuations. The cysts maintain an extremely low metabolism rate, remain in a state of obligate dormancy (diapause), and survive for prolonged periods of time. Diapause can be halted by specific environmental stimuli, which allows embryos to enter dormancy and post-embryonic development [25]. Post-embryonic development in *Artemia* cysts is divided into pre-emergent development, emergence, and larval development. During the emergence stage, mitochondrial maturation and yolk platelet degradation in a related manner, which results in the production of metabolites and enzymes that are essential for mitochondrial function [26] [28]. Vallejo et al. have shown that mitochondrial maturation in the absence of proliferation increases mitochondrial activity after the resumption of development in *Artemia* cysts [29]. The unique process of mitochondrial maturation provides us with a unique model in which mitochondrial biogenesis and metabolic regulation can be investigated.

In addition to mitochondria, yolk platelets are also important organelles involved in the regulation of metabolism during the resumption of development in *Artemia* cysts. Yolk platelets, which contain mainly proteins and lipids, are not static storage structures; instead, they have important roles in nauplius development and beyond. Yolk platelet degradation occurs in two phases. In the first non-enzymatic phase, the pH increases from 6.3 to 8.2, whereas in the second phase, yolk platelets fuse with lysosomes to initiate enzymatic degradation. This process releases enzymes and metabolites that are required for development [26]. Hand et al. have identified oxygen and intracellular pH as important factors in metabolic suppression in *Artemia* encysted embryos, which might occur via transcriptional repression in mitochondria [30] [31] [32]. However, the mechanism responsible for metabolic suppression is largely unknown.

In the present study, PHB1 was cloned and its expression during the resumption of development was characterized in *Artemia*. By RNA interference, PHB1 was found to have a role in mitochondrial maturation during post-embryonic development. A role for PHB1 in the degradation of yolk platelets was also found.

## Materials and Methods

### Animal culture and sample collection

*Artemia parthenogenetica* from Gahai Lake, China, was a gift from Professor Feng-Qi Liu (Nankai University, Tianjin, China). Specimens were separated into two random groups and cultured under different conditions. The first group was cultured in 8% artificial seawater (Blue Starfish, Hangzhou, Zhejiang, China) with a 5 h light cycle per day. Under these conditions, most *Artemia* reproduced oviparously and released encysted diapause embryos. The second group was cultured in 4% artificial seawater with a 19 h light cycle per day. Almost all Artemia in this group reproduced ovoviviparously and yielded swimming nauplii. Both groups were reared at 28°C and fed with Chlorella powder (Fuqing King Dnarmsa Spirulina Co. Ltd., Fuqing, China) every 2 days.

To obtain post-diapause (activated) embryos, cysts released by adult Artemia were dehydrated in a saturated sodium chloride solution for 24 h and then frozen at −20°C for 3 months. To obtain hatching samples, post-diapause cysts were hydrated for 5 h at 4°C and then incubated in 2% artificial seawater under continuous light at 25°C. Samples were taken at 0, 6, 12, and 24 h. All samples were snap-frozen in liquid nitrogen and stored at −80°C until use.

### Molecular cloning of PHB1

Total RNA was extracted using TRIzol reagent (Invitrogen, Carlsbad, CA, USA) according to the manufacturer's instructions. First-strand cDNAs were synthesized from 1 µg of total RNA by M-MLV reverse transcriptase (TaKaRa Bio, Shiga, Japan) in a reaction volume of 12.5 µl. The PHB1-encoding cDNA fragment was obtained through two rounds of polymerase chain reaction (PCR) amplification using degenerate primers (Table S1). To obtain the full-length PHB1 cDNA, 3′ and 5′ rapid amplification of cDNA ends was performed with gene-specific primers (Table S1) using the FirstChoice RLM-RACE kit (Ambion, Austin, TX, USA).

### Real-time polymerase chain reaction

Real-time PCR reactions were performed on the Bio-Rad MiniOpticon real-time PCR system using SYBR Premix Ex Taq (TaKaRa Bio, Shiga, Japan), and 200 nM of each specific primer (ATP6F, ATP6R, ND5F, ND5R, CytoBF, and CytoBR; Table S1) was used. Relative transcript levels were presented as fold-change, which was calculated using the comparative CT method as described by Livak and Schmittgen with α-tubulin as the internal reference [33].

### Antibody production

The open reading frame of the ArPHB1 gene was cloned (ExF and ExR, Table S1) into the pET-28 vector (Novagen) to generate the 6×His-PHB1 fusion protein. After purification using Ni-NTA resin and the QIAexpressionist kit (Qiagen, Germany), the protein was used to generate a polyclonal antibody against PHB1 (HuaAn, Hangzhou, China). For the phospho-PHB1 antibody, the phosphorated peptides (D(p)YEERVLPSITSEVL and DYEERVLP(p)SITSEVL) were used to generate a polyclonal antibody (HuaAn, Hangzhou, China). Serum was pre-cleared with the corresponding peptide that was not phosphorated before purification. The specificity of the anti-phospho-PHB1 antibody was tested by dot blotting (Fig. S2). In brief, peptides (0.04 and 0.004 µg) were applied directly onto a PVDF (polyvinylidene difluoride) membrane (Millipore Corp., Billerica, MA, USA) as dots and then detected with the corresponding antibody.

### Western blot analysis

After resolving proteins by 8% or 10% SDS-PAGE, proteins were transferred onto PVDF membranes. The membranes were incubated with primary antibodies overnight at 4°C, and proteins were visualized using the BM chemiluminescence western blotting kit (Roche Applied Science, Penzberg, Upper Bavaria, Germany). The ubiquitin antibody was purchased from Abcam (ab134953) (Cambridge, England, United kingdom).

### RNA interference

The double-stranded RNA was prepared as previously described using gene-specific primers (dsPHBF and dsPHBR; Table S1) and subcloned into the pET-T7 vector between Xho I and EcoR I sites. Plasmids expressing green fluorescent protein (GPF) dsRNA as the negative control were constructed as described. The recombinant plasmids were transformed into *Escherichia coli* HT115, and the dsRNA was produced and purified as described [28]. PHB and GFP dsRNAs (1 µg of each) were injected separately into specimens using the UltraMicroPump II system (World Precision Instruments Inc., Sarasota, FL, USA) equipped with a Micro4 microsyringe pump controller (World Precision Instruments Inc.). The injected specimens were cultured under previously described conditions to obtain ovoviviparous nauplii.

### Transmission electron microscopy analysis

Nauplii were fixed for 12 h in 2.5% glutaraldehyde prepared in phosphate-buffered saline (PBS). Thereafter, they were washed and post-fixed in 1% osmium tetroxide, dehydrated in a graded acetone series, and embedded in Spurr resin. Sections of 70 nm in thickness were obtained with a Leica EM UC6 microtome (Wetzlar, Germany), stained with 2% uranyl acetate and Reynold's solution (0.2% sodium citrate and 0.2% lead nitrate), viewed by a JEM-1230 transmission electron microscope (JEOL, Akishima, Tokyo, Japan), and photographed at a voltage of 70 kV.

### Isolation of yolk platelets and nuclei

Isolation of yolk platelets and nuclei was performed as previously described [34] using small volumes of buffers, which were supplemented with a protein inhibitor complex (Sangon Biotech, ShangHai, China) and 1 mM PMSF (Phenylmethanesulfonyl fluoride). In brief, approximately 100 mg of sample was homogenized using a glass homogenizer in 1 ml HM buffer (10 mM Tris−HCl, 10 mM NaCl, and 10 mM MgCl_2_, pH 7.5) containing 0.1% NP-40. The homogenate was filtered through a cell strainer (pore size, 38 µm) and centrifuged at 800×*g* for 10 min at 4°C. The pellet was gently resuspended in 200 µl of HM buffer, and the sample was layered onto a 1.2 ml Percoll layer (75% Percoll containing 150 mM NaCl, 10 mM MgCl_2_, and 10 mM Tris−HCl, pH 7.5) in a 2 ml centrifuge tube. The sample was centrifuged in a fixed-angle rotor at 15000×*g* for 15 min at 4°C. Nuclei accumulating at the sample/Percoll interface and yolk platelets sedimenting at the bottom were collected separately, washed with 5-volumes of HM buffer, and centrifuged at 850×*g* for 10 min at 4°C. Thereafter, yolk platelets were resuspended in RIPA lysis buffer (Beyotime, Shanghai, China), and the sample was maintained at 4°C for 30 min. The sample was then centrifuged at 12000×*g* for 5 min, the supernatant was collected, and stored at −80°C for immunoprecipitation. Nuclei were collected and fixed in 4% paraformaldehyde for immunofluorescence.

### Membrane and cytosol fraction

In yolk platelets and nuclei isolation, after 800×*g* centrifugation, the supernatant was subjected to further centrifugation at 20000×g. The pellet and supernatant was taken as membrane and non-membrane fractions respectively.

### Nuclear immunofluorescence

Nuclei were fixed in 4% paraformaldehyde overnight and then washed with PBS. Fixed nuclei were incubated in blocking buffer (0.3% Triton X-100 and 5% bovine serum albumin in PBS) for 1 h at room temperature. Thereafter, nuclei were labeled with an anti-PHB antibody in 0.3% Triton X-100 and 3% bovine serum albumin in PBS overnight at 4°C, followed by incubation in a secondary Alexa Fluor 488 donkey anti-rabbit antibody (Invitrogen) for 1 h at room temperature. DNA was stained by DAPI (4′,6-diamidino-2-phenylindole dilactate) for 10 min at room temperature. Then stained nuclei were visualized by a LSM 710 confocal microscope (Jena, Germany).

### Immunoprecipitation

For immunoprecipitation, yolk platelet lysate (500–1000 µg) and an anti-PHB1 antibody (10 µg) were incubated with gentle rocking overnight at 4°C. Thereafter, protein A agarose beads (20 µl of a 50% bead slurry, Millipore Corp.) were added, and samples were incubated with gentle rocking for 1 h at 4°C. After centrifugation for 30 sec at 4°C, the pellet was washed five times with 100 µl of RIPA lysis buffer. The immunocomplex was eluted by adding SDS loading buffer (4% SDS, 0.2% bromophenol blue, 20% glycerol, and 200 mM β-mercaptoethanol) and boiled in a water bath for 5 min.

### Limited trypsin digestion assay

The immunoprecipitation product, other than eluted by SDS loading buffer,from the protein A agarose beads, was washed twice with PBS. Then the the protein A beads with the immunoprecipitates were incubated with trypsin (0.05% in 0.53 mM EDTA; Corning, VA, USA) at an enzyme to substrate ratio of 1∶25 (w/w) at 30°C for 30 min. The digest was then subjected to Western blot analysis as above by using anti-ubiquitin (Abcam, ab134953) or anti-PHB1 antibody. The intact immunoprecipitated products served as controls.

### AICAR treatment

As a cell permeable activator of AMP-activated protein kinase (AMPK), 5-Aminoimidazole-4-carboxamide 1-β-D-ribofuranoside Acadesine N1-(β-D-Ribofuranosyl)-5-aminoimidazole-4-carboxamide (AICAR) (Sigma, 3050 Spruce St., St. Louis, Missouri 63103, United States) was used to activate AMPK. In the NM treatment experiment, rehydrated post-diapause embryos were incubated in 2% seawater containing 0.5 and 1 mM for 24 h. After that the nauplii were collected for Western blotting analysis.

## Results

**Figure 1:**
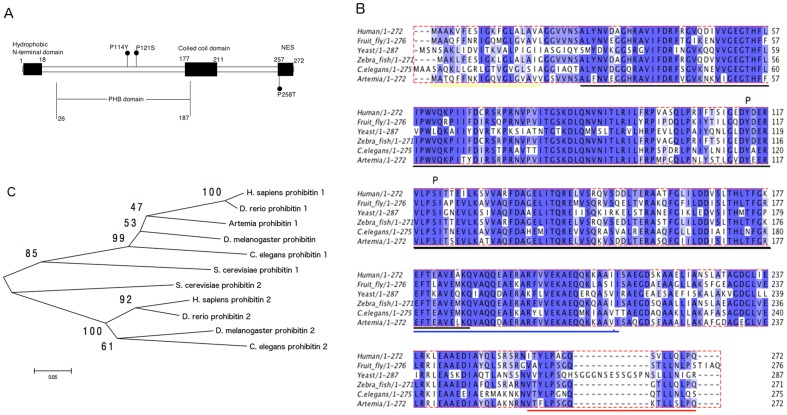
Sequence and phylogenetic analysis of ArPHB1. A, Schematic view of PHB1 architecture. B, Amino acid sequence alignment of ArPHB1 with PHB1 from other species. The sequences used in this alignment and their Genbank/EMBL/DDBJ or SWISS-PROT database accession numbers are listed in Table S2. Gaps inserted to maximize alignment are denoted by hyphens. The amino acid position numbers are shown to the right of the sequences. The phosphorylation sites against which the antibody was generated is marked by P. Conserved domains are marked by lines below sequences (the N-terminal hydrophobic domain by yellow, the PHB domain by black, the coiled-coil domain by blue, and the nuclear export sequence by red). The alignment was performed by MEGA5 using the ClustalW method. C, A phylogenetic tree of the amino acid sequences of prohibitins. The sequences used in this analysis and their GenBank/EMBL/DDBJ or SWISS-PROT database accession numbers are listed in Table S1. The phylogenetic tree was constructed using the Neighbor-joining method. Bootstrap percentage values for 1000 replicate analysis are shown at branching points. The bar at the bottom shows the branch length, and it corresponds to the mean number of differences (0.05) per residue along each branch.

To investigate the role of ArPHB1 in the development of *Artemia*, a 988-bp cDNA encoding ArPHB1 was cloned by PCR from post-diapause embryos of *A. parthenogenetica* from Gahai Lake using DF1, DF2, DR1, and DR2 primers for degenerate PCR and 3F1, 3F2, 5R1, and 5R2 primers for the rapid amplification of cDNA ends (Table S1). Sequence analysis revealed the cDNA to contain an open reading frame encoding a protein of 272 amino acids with a predicted mass of 29.9 kDa (Fig. S1). The deduced amino acid sequence showed that ArPHB1 had a conserved prohibitin domain, a coiled-coil domain, an N-terminal hydrophobic sequence, and a nuclear export sequence (NES) (Fig. 1A). ArPHB1 was homologous to PHB1 in other species. For example, sequence alignment showed 78% sequence identity with fruit fly PHB1, 74% with zebrafish PHB1, 68% with nematode PHB1, 74% with human PHB1, and 50% with yeast PHB1 (Fig. 1B). Phylogenetic analysis confirmed ArPHB1 to derive from PHB1 (Fig. 1C).

**Figure 2:**
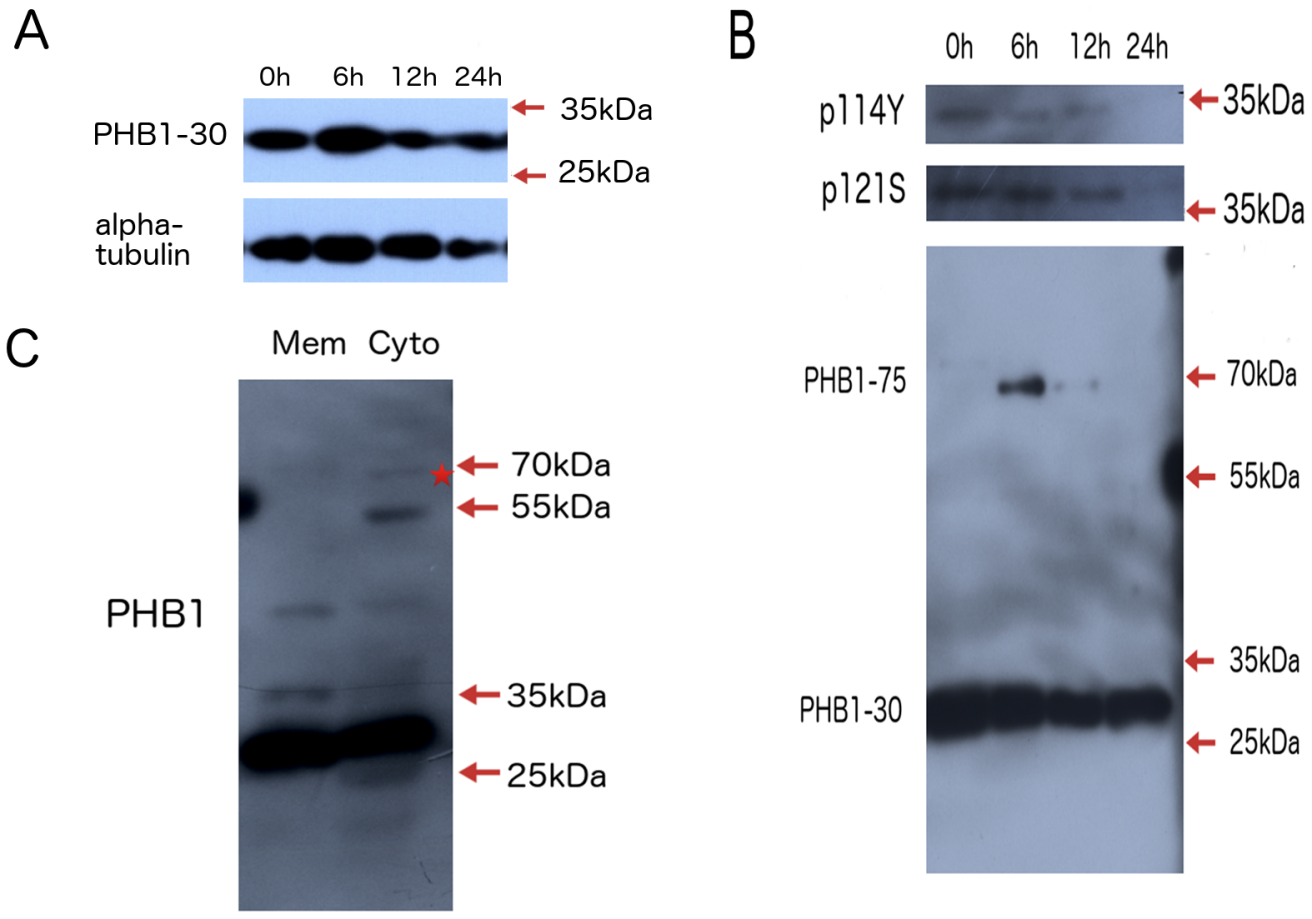
Characterization of ArPHB1 during post-embryonic development. A, Western blot analysis of PHB1. 0 h, 0 h-incubated cysts; 6 h, 6 h-incubated cysts; 12 h, 12 h-incubated cysts; 24 h, 24 h-incubated nauplius. B, Western blot analysis of phosphorylated PHB1 and PHB1 modifications. The same samples were loaded in B as in A. C, PHB1-75 existed as a non-membrane form of PHB1 in *Artemia*. Mem, membrane fraction obtained by 20000×g centrifugation after nuclei isolation (800×g, 10 min); Cyto, cytosol fraction obtained after 20000×g centrifugation. PHB1-75 was marked by an asterisk.

To characterize ArPHB1, an antibody was produced using full-length ArPHB1 after the protein was expressed in *E. coli*. Western blot analysis showed two bands of 30 kDa (predicted molecular weight) for PHB1-30 and 75 kDa for PHB1-75. The major protein band, PHB1-30, showed no significant changes in expression (Fig. 2A), while PHB1-75 was observed during pre-emergent development (6 and 12 h incubation) (Fig. 2B) when the embryos were preparing for energy- and nutrient- demanding processes (i.e., emergence and larval development). Through subcellular fraction we found that PHB1-75 existed as a non-membrane form of PHB1 in *Artemia* (Fig. 2C), which is consistent with previous report that the PHB1-75 existed in non-membrane fraction [27].We also produced antibodies against phosphor-Tyr-114 and phosphor-Ser-121 using respective peptides, and their specificities were tested by dot blotting (Fig. S2). The phosphorylation status of Tyr-114 and Ser-121, which are thought to be involved in metabolic control, were investigated. Both PHB1-114Y (35 kDa) and PHB1-121S (38 kDa) were phosphorylated in embryos during pre-emergent development (0, 6, and 12 h after incubation), but not in the nauplius (24 h after incubation) (Fig. 2B). These results indicate that PHB1 phosphorylation has roles in cell proliferation and metabolic control during post-embryonic development of *Artemia*.

**Figure 3:**
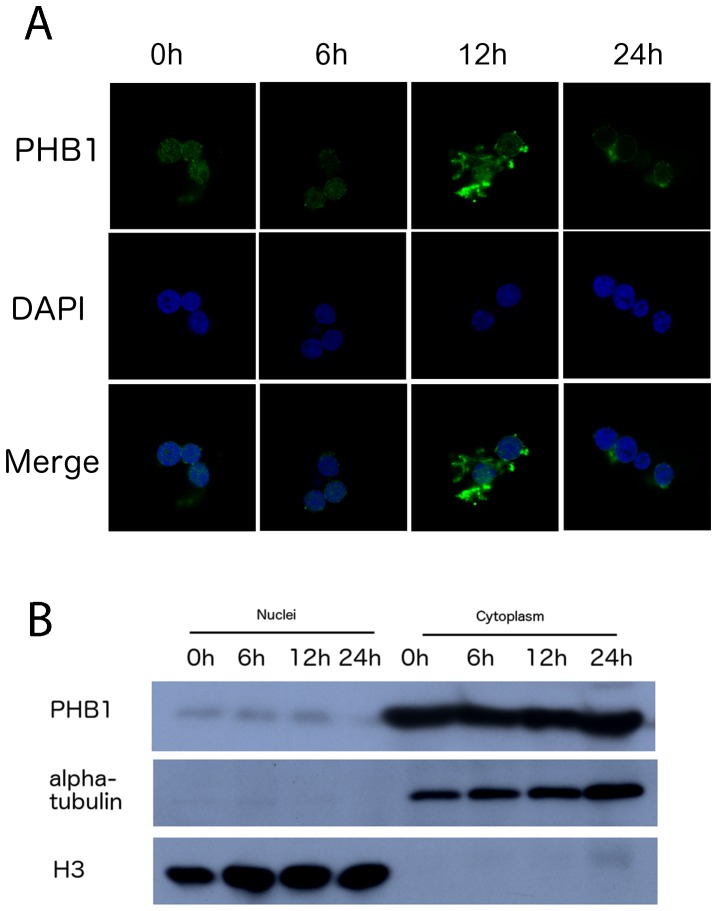
The localization of PHB1 in nuclei during *Artemia* post-embryonic development. A, Immunofluorescence analysis of the nucleus. Nuclei from 0, 6, 12, and 24 h-incubated nauplii were isolated and stained with an anti-PHB1 antibody. DNA was stained with DAPI (4,6-diamidino-2-phenylindole dilactate). B, Nuclei from the respective developmental stages were isolated and analyzed by Western blotting (anti-PHB1).

To explore whether the nuclear localization of PHB1 was also developmentally regulated, we localized PHB1 by immunofluorescence. PHB1 was found in the nuclear matrix during pre-emergent development (0, 6, and 12 h after incubation), but not 24 h after incubation (Fig. 3A), which was further confirmed by Western blotting analysis (Fig. 3B). Although PHB1 was exported out of the nuclear matrix, it remained at the nuclear membrane (Fig. 3A), illustrating that the localization of PHB1 was developmentally-related. These changes may be related to cell cycle progression or metabolic remodeling during the emergence stage.

**Figure 4:**
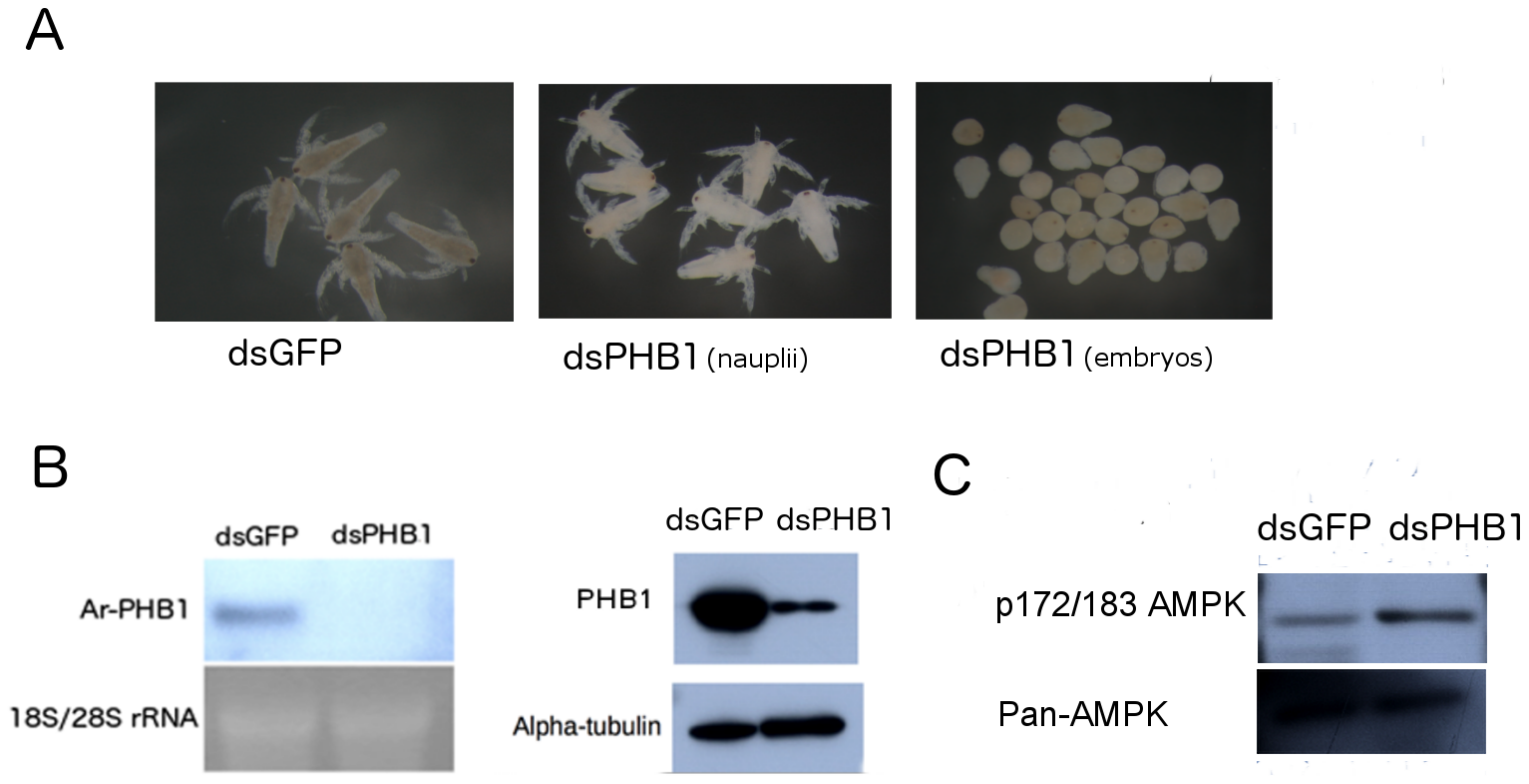
ArPHB1 knockdown resulted in the production of metabolic stress. A, Released nauplii were fixed in absolute ethanol and photographed under a dissecting microscope. dsGFP, dsGFP injected as control; dsPHB1(nauplii), nauplii released after ArPHB1 knockdown; dsPHB1(embryos), embryos released after ArPHB1 knockdown. B, Northern and western blot analyses to verify the efficiency of PHB1 knockdown. C, AMPK activity (p172/183) was investigated by western blot analysis in PHB1 knockdown and control groups.

RNAi was performed by microinjecting dsRNA into female embryos, which had oocytes in the oviducts. PHB1 knockdown resulted in the death of embryos or the production of swimming nauplii (Fig. 4A). Swimming nauplii released by the PHB1 knockdown group were sluggish compared to nauplii released by the control group (Video S1, S2), indicating that there were defects in energy homeostasis. Nauplii released by the PHB1 knockdown group were also weaker than those released by the control group (Fig. 4A). Northern and western blotting analyses showed successful RNAi knockdown (Fig. 4B).

**Figure 5:**
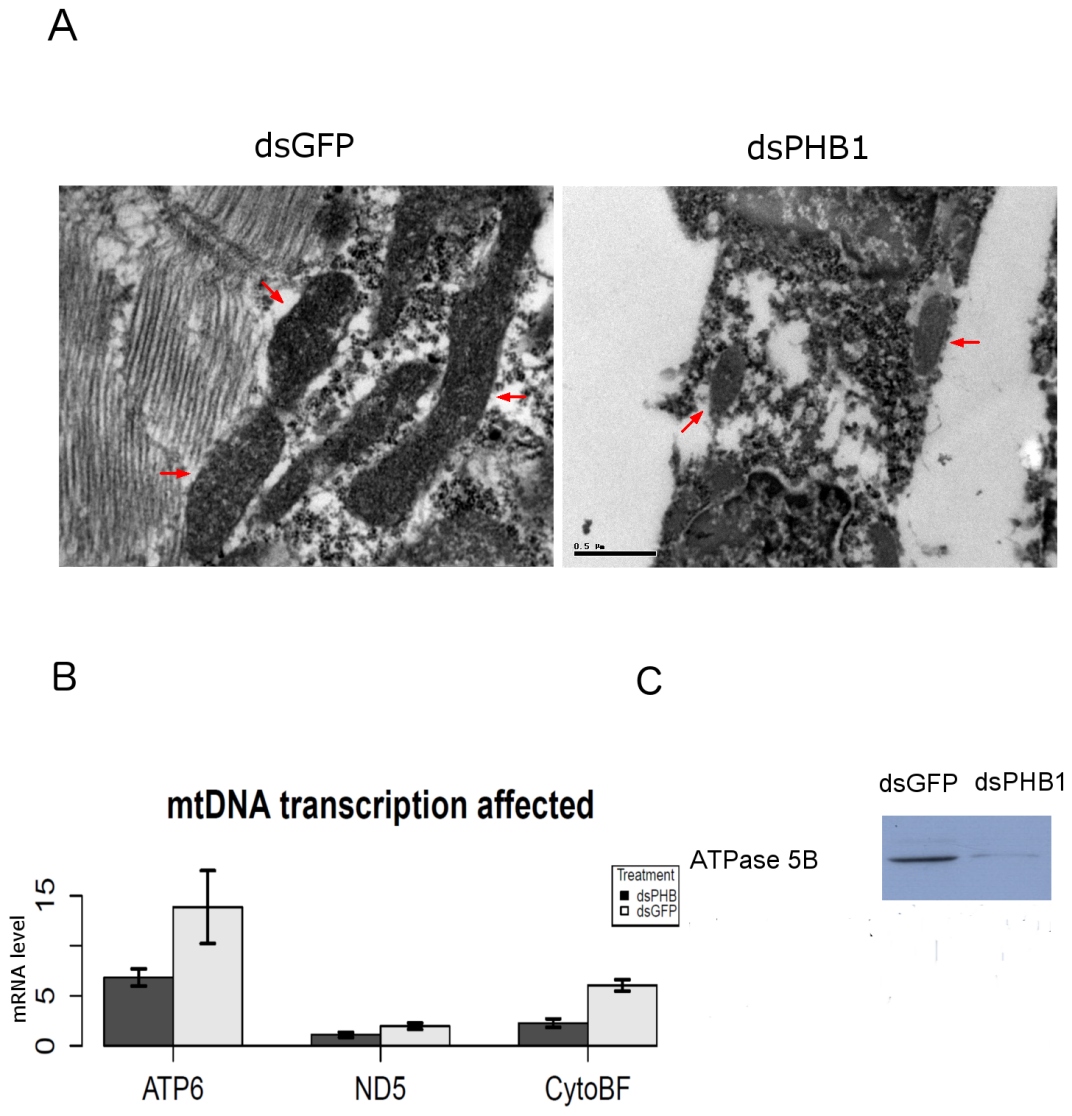
ArPHB1 knockdown resulted in aberrant mitochondrial function. A, mitochondrial morphology by TEM (magnification, 24000×; scale bar = 0.5 µm). Mitochondria are denoted by red arrows. B, Real-time PCR of mitochondrial genes (ATP6, ATP synthase F0 subunit 6; ND5, NADH dehydrogenase 5; and Cyto B, cytochrome b). Results were normalized to α-tubulin. Means ± SD are plotted. P<0.05. The primers used for real-time PCR are listed in Table S1. C, Western blot analysis of ATPase β subunit.

To investigate if PHB1 affected energy homeostasis, nauplii released at 1–1.5 day were collected. Western blot analysis illustrated an increase in AMPK activity (Fig. 4C). Thereafter, we investigated the role of PHB1 in mitochondrial function by RNAi. PHB1 knockdown adversely affected the morphology of mitochondria (Fig. 5A), which were smaller and more compact than those in nauplii released by the control group. RT-PCR analysis showed downregulation of mtDNA-encoded mRNAs in the PHB1 knockdown group (Fig. 5B), confirming the morphological defects in mitochondria. Moreover, western blot analysis revealed that F-ATPase β subunit, the enzyme for ATP synthesis, was also downregulated after PHB1 knockdown (Fig. 5C). These results indicate that PHB1 maintains normal mitochondrial biogenesis during the development of *Artemia*.

**Figure 6:**
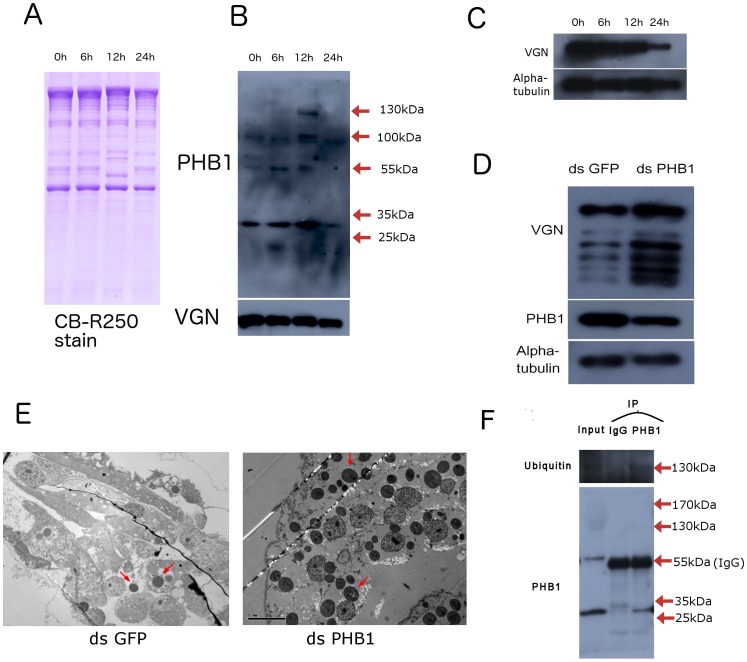
Involvement of PHB1 in the degradation of yolk platelets. A, SDS-PAGE analysis of isolated yolk platelet lysates. Proteins were stained by Coomassie brilliant blue R-250. 0 h, 0 h after incubation; 6 h, 6 h after incubation; 12 h, 12 h after incubation; 24 h, 24 h after incubation. B, Yolk platelets were isolated by density centrifugation and probed with an anti-PHB1 antibody. An anti-VGN antibody was used as the control. 0 h, 0 h after incubation of cysts; 6 h, 6 h after incubation of cysts; 12 h, 12 h after incubation of cysts; 24 h, 24 h after incubation of nauplii. C. Yolk protein degradation during embryonic development. 0 h, 0 h after incubation of cysts; 6 h, 6 h after incubation of cysts; 12 h, 12 h after incubation of cysts; 24 h, 24 h after incubation D, Western blot analysis of yolk protein degradation in PHB1 knockdown nauplii. E, TEM analysis showed aberrant yolk platelet degradation in the PHB1 knockdown group (magnification 1250×, scale bar = 1 µm). F, Immunoprecipitation with an anti-PHB1 antibody using isolated yolk platelet lysates (12 h after incubation of cysts). The precipitates were then probed with an anti-ubiquitin antibody.

Yolk platelets, which contain mainly yolk protein, are important organelles in metabolism. The degradation of yolk proteins is critical for the normal development of *Artemia* because the process produces nutrients and necessary enzymes [26]. Because PHB1 knockdown yielded a nutrient-deficient phenotype, we investigated whether PHB1 knockdown could affect the degradation of yolk proteins. We used an antibody against peptide located at the N-terminal of vitellogenin (anti-VGN), which was previously used by our lab to analyze the enzyme cleavage pattern of yolk proteins [35], to examine yolk platelets degradation. In this study, we isolated yolk platelets from post-embryonic developmental samples (0, 6, 12, and 24 h after incubation) (Fig. 6A). Yolk proteins in yolk platelets showed different bands at 12 h after incubation which may suggest initiation of enzymic degradation of yolk proteins in yolk platelets. Western blot analysis showed that PHB1 existed in yolk platelets, and the amount of PHB1 gradually increased during pre-emergent development and decreased in nauplii (Fig. 6B). In addition, several proteins bands were observed after using the anti-PHB1 antibody. Two of these protein bands electrophoresed at 130 and 170 kDa at 12 h after incubation (Fig. 6B), indicating that PHB1 has a role in yolk protein degradation during post-embryonic development. And the degradation of yolk protein is consistent with the amount of ArPHB1 in yolk platelets (Fig. 6C). In agreement with these results, PHB1 knockdown resulted in the accumulation of yolk proteins in nauplii (Fig. 6D). TEM analysis found that yolk platelet degradation was blocked in nauplii after PHB1 knockdown, which might have caused nutrient deficiency (Fig. 6E). These results illustrate that PHB1 is indispensable for yolk platelet degradation during the early development of *Artemia*.

Attachment of ubiquitin molecules can target organelles (peroxisomes) to lysosomes for degradation [36]. To determine whether the two additional protein bands at 12 h after incubation were ubiquitinated PHB1, PHB1 in yolk platelet lysates was immunoprecipitated with an anti-PHB1 antibody. The 130 kDa protein band was found to be ubiquitinated PHB1 (Fig. 6F). These results illustrate that PHB1 may mediates yolk platelets degradation via ubiquitination.

## Discussion

In response to unfavorable environmental conditions, *Artemia* produce encysted gastrula embryos in diapause, a state of obligate dormancy, in which complex metabolic processes are downregulated to minimize cellular activities and conserve resources. Organisms in diapause remain hypometabolic, even under conditions that would normally promote active metabolism and development. In this state, organisms in diapause are more tolerant of environmental stresses. In addition, a greater metabolic arrest usually associates with a longer dormant state [37]. During post-embryonic development, metabolic suppression ceases to meet energy and nutrient needs for normal development, providing us with a unique model in which metabolic remodeling can be studied.

The control of energy storage in organisms in diapause is an emerging area of research, which may provide insights on the aging process and age-related diseases because there are crosstalk between diapause and aging. A recent study has shown the metabolic and stress sensor, AMP-activated protein kinase (AMPK), directly suppress adipose triglyceride lipase (ATGL-1) activity by phosphorylation, which reserves lipids in the dauer of *C. elegans* [38]. And appropriate level of ROS was also important for reserving lipids in the dauer of *C. elegans* through hypoxia inducible factor-1 (HIF-1) signalling [39]. It is reported that PHBs modulate fat content and mitochondrial metabolism in *C. elegans* [15]. According to previous report that the ubiquitin of protein and metabolic state in dormancy cyst is reversibly regulated in response to anoxia [25] [40], our results here suggests that keep ArPHB1 from ubiquitination may be a mechanism to reserve the yolk platelet in dormancy cysts.

In this study, we investigated the expression of ArPHB1 during post-embryonic development of *Artemia* when encysted embryos resume their metabolism to meet developmental requirements [26]. The expression of ArPHB1 during post-embryonic development was relatively constant because ArPHB1 is an important scaffolding protein involved in several biological processes. In characterization of PHB1 expression during embryonic development, a band of 75 kDa, which was analysed in a previous paper as a non-membrane form of PHB1 (27), was detected after 6 h of hatching and gradually disappeared after 12 h incubation. After 6 h incubation the encysted embryos have resumed respiration activities, the appear of a non-membrane-attached form of PHB1 may respresent the tranportation of ArPHB1 in the cell, which might lead to the increase of ArPHB1 in yolk platelet. There is a strong association between the regulation of PHB1, and its modifications and cellular localizations. There are changes in the phosphorylation status and localization of ArPHB1 during emergence, which marks the initiation of cell proliferation and the increased activities of many enzymes [26] [28]. Given that cell proliferation requires amino acids, nucleotides, and energy, PHB1 may mediate metabolic remodeling to meet the needs of cell proliferation and development. It is also possible that ArPHB1 directly regulates cell proliferation during post-embryonic development.

ArPHB1 knockdown increased AMPK activity in nauplii. Thus, we used AICAR, an activator of AMPK, to activate AMPK; however, AICAR did not affect swimming or cold resistance in nauplii from the knockdown group (data not shown). By contrast AICAR treatment accelerated yolk degradation when the anti-VGN antibody was used for western blot analysis (Fig. S3). Therefore, we conclude that the increase in AMPK activity was not responsible for the defects in nauplii caused by ArPHB1 knockdown. The increase in AMPK activity after ArPHB1 knockdown was most likely caused by damaged mitochondria. Although an increase in AMPK activity usually associates with the degradation of yolk protein, ArPHB1 knockdown blocked this effect.

Yolk degradation is critical in the cascade of events leading to emergence [26]. It has been reported that AMPK homolog in *C. elegans* (aak-2) affected the role of PHB1 in fat metabolism [15]. However, this does not apply to the degradation of yolk proteins. Our results showed that PHB1 participate directly in the degradation of yolk platelets independent of AMPK activity. Due to the highly conserved amino acid and functional domains, the involvement of PHB1 in yolk degradation is likely to be conserved in other organisms. And PHB1 may serve as substrates of ubiquitination in yolk platelets that target the yolk platelets for selective autophagy. Besides, a tryptic digestion of the anti-PHB1 IP product resulted in a band of about 37 kDa, indicating that the 130-kDa molecule was the ubiquitinated form of PHB1 (Fig. S4).

In the ArPHB1 knockdown experiment, 50% of the embryos were released as swimming nauplii and the remaining 50% were released as dead embryos (Fig. 4A). The blockage of degradation of yolk platelets caused malnutrition may be the cause of the weaker nauplii in the ArPHB1 knockdown group. The different fates of the embryos may be due to PHB1 knockdown at different stages of oocyte development. For example, the dsRNA was injected when oocytes were in the oviduct, and oocytes were not developmentally synchronized. These results show that ArPHB1 has other lethal roles in embryonic development.

Our results illustrate that PHB1 is critical for post-embryonic and early larval development of *Artemia*. PHB1 regulates mitochondrial maturation and mediates yolk platelet degradation, which support high energy cellular activities during post-embryonic and larval development. Our results also suggest that PHB1 functions in yolk platelet degradation as substrates of ubiquitination in yolk platelet.

## Supporting Information

Figure S1 **The ArPHB1 cDNA sequence and its deduced amino acid sequence.**
(TIF)

Figure S2 **Antibody specificities for phosphor-Tyr-114 and phosphor-Ser-121 of PHB1.** The peptides were applied directly onto PVDF membranes as dots and then detected with the corresponding antibody.
(TIF)

Figure S3 **The effect of AICAR, an AMPK activator, on the degradation of yolk platelet proteins.** The encysted embryos were incubated at 2% seawater containing 0, 0.5, and 1 mM AICAR for 24 h. For each lane, 7 nauplius were homogenized in SDS loading buffer (4% SDS, 0.2% bromophenol blue, 20% glycerol, and 200 mM β-mercaptoethanol) and loaded.
(TIF)

Figure S4 **Tryptic digestion and Western blotting analysis of PHB1 IP product.** 1 and 2, Western blotting of intact (1) and digested (2) IP product by anti-ubiquitin; 1′ and 2′, Western blotting of intact (1′) and digested (2′) IP product by anti-PHB1. The 130- and 37-kDa bands are indicated by red and blue asterisks respectively.
(TIF)

Table S1 **Nucleotide sequences of primers used in polymerase chain reactions.**
(DOCX)

Table S2 **Protein sequences used in the alignment of sequences and phylogenetic analysis.**
(DOCX)

Video S1 **Nauplii reproduced by the adults, into which fragment of GFP dsRNA was microinjected as control.**
(AVI)

Video S2 **Nauplii reproduced by the adults, into which fragment of ArPHB1 dsRNA was microinjected.**
(AVI)
