# Supplementary figures and images for: Characterization of PHB1 and Its Role in Mitochondrial Maturation and Yolk Platelet Degradation during Development of Artemia Embryos
Source: PLoS One. 2014 Oct 13;9(10):e109152. doi: 10.1371/journal.pone.0109152 (PMC4195616; doi:10.1371/journal.pone.0109152)

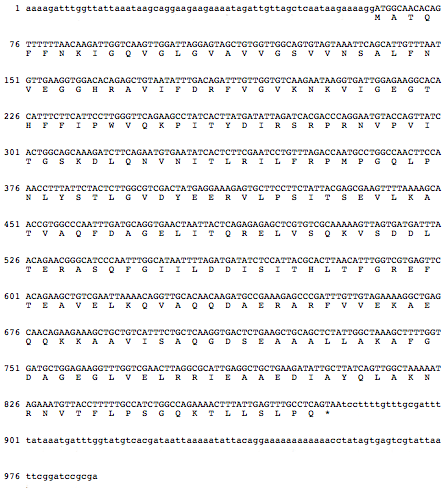

Supplement: Figure S1 — The ArPHB1 cDNA sequence and its deduced amino acid sequence. (TIF) [file pone.0109152.s001.tif]

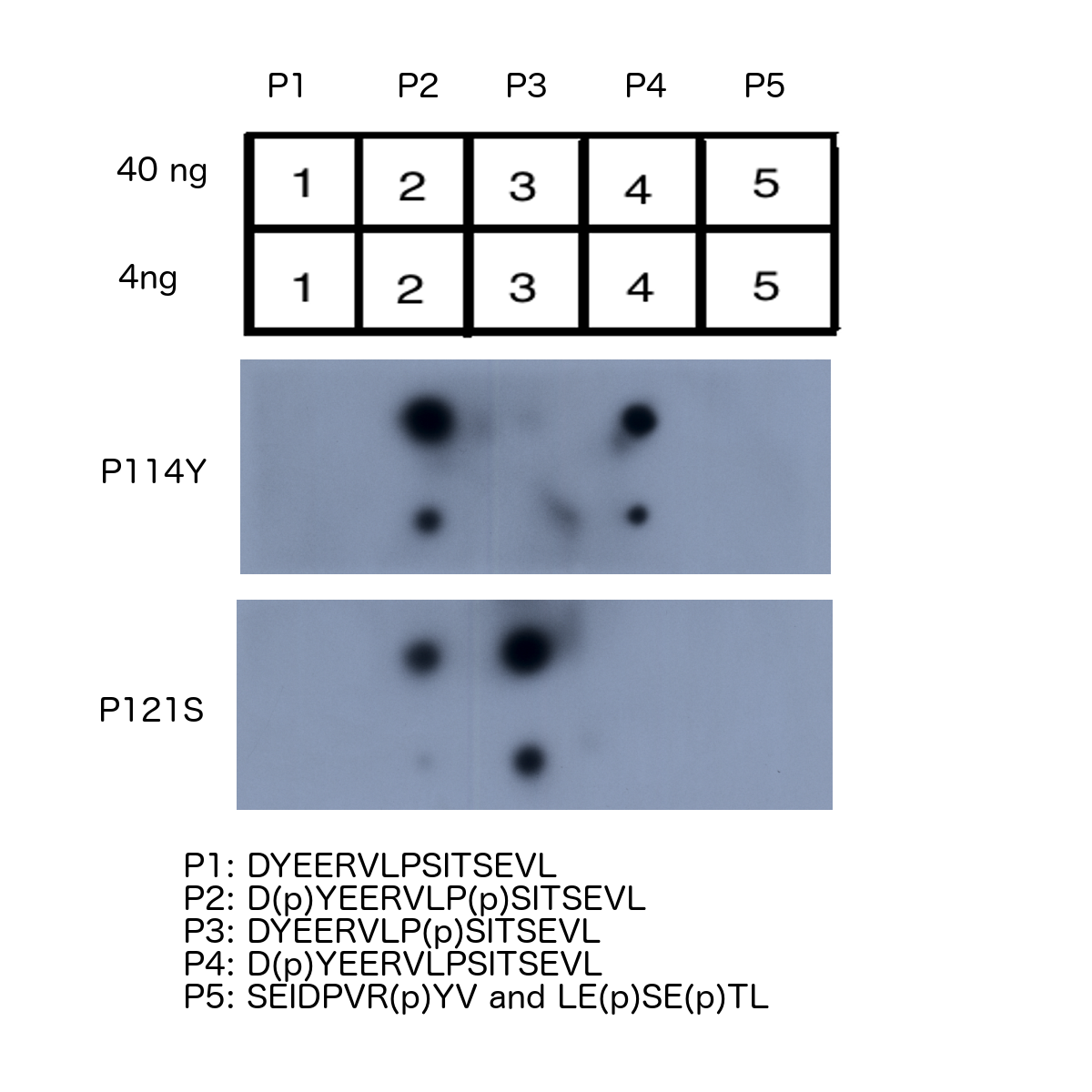

Supplement: Figure S2 — Antibody specificities for phosphor-Tyr-114 and phosphor-Ser-121 of PHB1. The peptides were applied directly onto PVDF membranes as dots and then detected with the corresponding antibody. (TIF) [file pone.0109152.s002.tif]

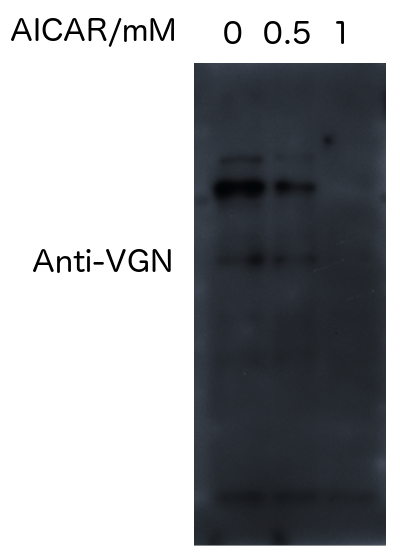

Supplement: Figure S3 — The effect of AICAR, an AMPK activator, on the degradation of yolk platelet proteins. The encysted embryos were incubated at 2% seawater containing 0, 0.5, and 1 mM AICAR for 24 h. For each lane, 7 nauplius were homogenized in SDS loading buffer (4% SDS, 0.2% bromophenol blue, 20% glycerol, and 200 mM β-mercaptoethanol) and loaded. (TIF) [file pone.0109152.s003.tif]

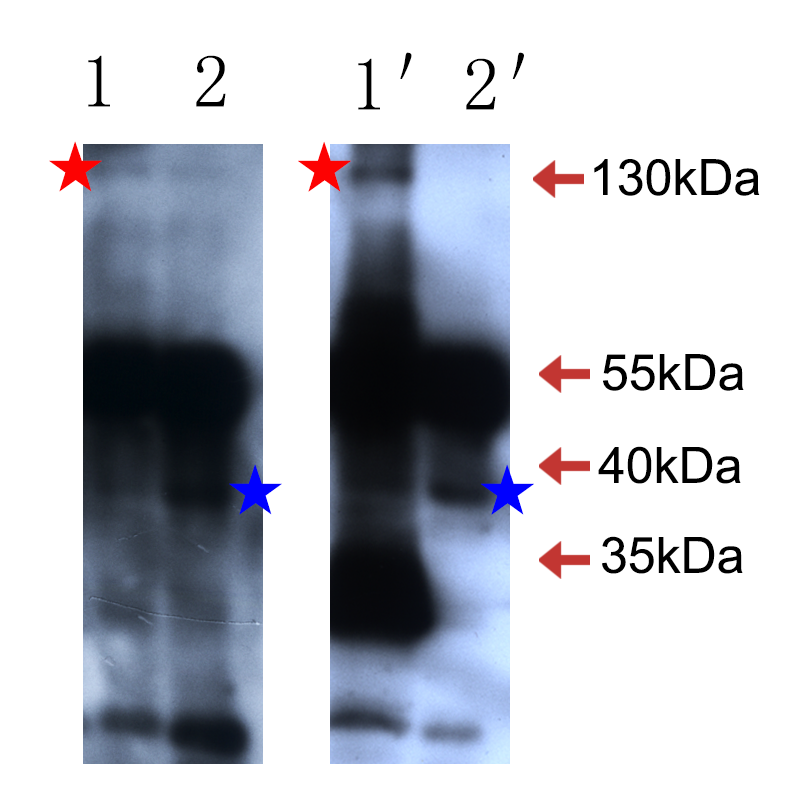

Supplement: Figure S4 — Tryptic digestion and Western blotting analysis of PHB1 IP product. 1 and 2, Western blotting of intact (1) and digested (2) IP product by anti-ubiquitin; 1′ and 2′, Western blotting of intact (1′) and digested (2′) IP product by anti-PHB1. The 130- and 37-kDa bands are indicated by red and blue asterisks respectively. (TIF) [file pone.0109152.s004.tif]
