# Supplementary material for: Characterization of PHB1 and Its Role in Mitochondrial Maturation and Yolk Platelet Degradation during Development of Artemia Embryos
Source: PLoS One. 2014 Oct 13;9(10):e109152. doi: 10.1371/journal.pone.0109152 (PMC4195616; doi:10.1371/journal.pone.0109152)
Supplement: Table S1 — Nucleotide sequences of primers used in polymerase chain reactions. (DOCX) [file pone.0109152.s005.docx]

Table S1. Nucleotide sequences of primers used in polymerase chain reactions

| **Primer** | **Length(bp)** | **Direction** | **Sequence(5’-3’)** |
| --- | --- | --- | --- |
| **DF1** | 20 | F | TGGRTHCARMRNCCNATCAT |
| **DF2** | 19 | F | TDCARAMHGTNAAYATYAC |
| **DR1** | 20 | R | TTSWBYTGYTCNGCYTTYTC |
| **DR2** | 20 | R | TCYTGYTGNGCNACYTGYTT |
| **5’R1** | 24 | R | TGGGATGCCCGTTCTGTTAAATCA |
| **5’R2** | 30 | R | CAAGAGTAGAATAAAGGTTTGGAAGTTGGC |
| **3’F1** | 23 | F | CACGACCCAGGAATGTACCAGTT |
| **3’F2** | 30 | F | GCCAACTTCCAAACCTTTATTCTACTCTTG |
| **dsPHBF** | 30 | F | GCTCGAGAGATCATTTATGATATTAGATCA |
| **dsPHBR** | 31 | R | CCGGAATTCCTTGTTTTAATTCGACAGCTTC |
| **ExF** | 30 | F | CGCGGATCCATGGCAACACAGTTTTTTAAC |
| **ExR** | 31 | R | CCGCTCGAGCAAACAAAAGGATTACTGAGGC |
| **ATP6F** | 22 | F | TAAACCGAGAAATGTCCCTGCT |
| **ATP6R** | 22 | R | ATAGGACTGGATGACAGCGACA |
| **ND5F** | 22 | F | GCGAGGATAAGCAAGAAACACC |
| **ND5R** | 22 | R | TTAGAGCGGTGGCTTTATGTTG |
| **CytoBF** | 22 | F | CGTGGGCTTTATGATTCTTGTC |
| **CytoBR** | 22 | R | GTCGTGCTCCGATTCAAGTAAG |

F and R indicate the forward and reverse directions, respectively.
