# Supplementary material for: Characterization of PHB1 and Its Role in Mitochondrial Maturation and Yolk Platelet Degradation during Development of Artemia Embryos
Source: PLoS One. 2014 Oct 13;9(10):e109152. doi: 10.1371/journal.pone.0109152 (PMC4195616; doi:10.1371/journal.pone.0109152)
Supplement: Table S2 — Protein sequences used in the alignment of sequences and phylogenetic analysis. (DOCX) [file pone.0109152.s006.docx]

Table S2. Protein sequences used in the alignment of sequences and phylogenetic analysis

| **Organisms/protein** | **Uniprot ID/Gene ID** |
| --- | --- |
| **Human/prohibitin 1** | P35232 |
| **Fruit fly/prohibitin 1** | P24156 |
| **Yeast/prohibitin 1** | P40961 |
| **Zebra fish/prohibitin 1** | Q7T1D8 |
| ***C. elegans*/prohibitin 1** | Q9BKU4 |
| **Human/prohibitin 2** | Q99623 |
| **Fruit fly/prohibitin 2** | Q9VZA4 |
| ***C. elegans*/prohibitin 2** | P50093 |
| ***D. rerio*/prohibitin 2** | gi350276142 |
| ***S. cerevisiae*/prohibitin 2** | P50085 |
